# ‘We need to make “shit” sexy’ a qualitative study exploring treatment adherence in adolescents with inflammatory bowel disease

**DOI:** 10.1080/21642850.2025.2500323

**Published:** 2025-05-05

**Authors:** Cassandra Screti, Lou Atkinson, Rachel Shaw, Rafeeq Muhammed, Gemma Heath

**Affiliations:** a Institute of Health & Neurodevelopment, Aston University, Birmingham, UK; b School of Health and Life Sciences, Aston University, Birmingham, UK; c Birmingham Women’s, and Children’s Hospital, Birmingham, UK

**Keywords:** Inflammatory bowel disease, adolescence, paediatrics, treatment adherence, medication adherence

## Abstract

**Background:** Adolescents with inflammatory bowel disease (IBD) are faced with the complexities of acquiring self-management behaviours at a time when they are also navigating developmental challenges associated with adolescence. To date, limited treatment adherence interventions exist to support adolescents with IBD.

**Aim:** To explore the experience and support needs of adolescents with IBD to facilitate optimum treatment adherence.

**Method:** Thirty-three semi-structured interviews were conducted with adolescents with IBD (*n* = 12), parents of adolescents with IBD (*n* = 13) and healthcare professionals who support adolescents with IBD (*n* = 8). Adolescents and parents completed a creative task to prioritise adherence barriers and adherence intervention strategies.

**Results:** The analysis generated three key themes: (1) striving for normality, (2) taking responsibility for IBD management and (3) seeking supportive environments. Living with IBD was often perceived as living a limited life, as adolescents had to manage their symptoms, which resulted in feelings of difference and stigmatisation. To manage their IBD, adolescents were required to develop treatment routines and communicate their health needs. Parents wanted to protect their child from the burden of living with IBD. Synthesis of findings with a creative mapping task generated seven priorities for intervention.

**Discussion:** Adolescents discussed the complexity behind their adherence behaviours and the formation of treatment perceptions. The adherence barriers identified within this research can be utilised to develop a treatment adherence intervention that is effective for adolescents with IBD.

## Background

Inflammatory Bowel Disease (IBD) is a collective term used to describe Crohn’s Disease, Ulcerative Colitis and IBD-Unknown. All involve inflammation of the digestive system and are characterised by periods of symptom relapse and remittance (Yu & Rodriguez, 2017). Symptoms can include pain, fatigue, diarrhoea, bloating and cramping (Singh et al., 2011). Approximately 25 per 100,000 young people aged 10–16, in the UK, live with IBD (Pasvol et al., 2020). However, incidences of IBD are growing among adolescents worldwide (Kuenzig et al., 2022; Ye et al., 2020). Treatment plans for IBD are unique to the individual and contain a combination of medication routines and lifestyle behaviour changes (Ananthakrishnan et al., 2022; Hanghoj & Boisen, 2014).

Rates of medication non-adherence in adolescents with IBD are reported to be as high as 65–93% (Knowles & Alex, 2020; Spekhorst et al., 2016). Non-adherence presents in several forms, including missing occasional doses, lowering, or increasing the dose or failing to administer all the prescribed doses (Horne & Weinman, 1999). Non-adherence can be volitional (an intentional choice) and/or non-volitional, (an unintentional consequence of adherence barriers). Volitional non-adherence is attributed to multiple factors including medication satisfaction, wanting to feel more in control, feeling well, greater disease activity and having a poorer quality of life (QoL) (Schurman et al., 2011). IBD treatment aims to lower disease activity and maintain symptom remittance, meaning adolescents are required to administer medications when they feel well (Sandhu, 2007). As adolescents can find it difficult to consider the long-term or unseen consequences of current behaviour, those with IBD may be more inclined to not adhere to treatment when there are no immediate repercussions (Taddeo et al., 2008).

Theoretical models further our understanding of non-adherence as a health behaviour. The necessity and concerns framework (NCF) (Horne & Weinman, 2002) suggests adherence is related to an individual’s beliefs about the necessity of their treatment, as well as concerns about adverse side effects of treatment. Medication beliefs have been identified as a primary reason for non-adherence in adults with IBD (Jackson et al., 2010). Due to the asymptomatic nature of IBD, adolescents may not believe they need to adhere to their treatment plan during periods of wellness. Adolescents may also be less concerned about the long-term consequence of non-adherence due to their present mindedness. In addition, different IBD medications may induce different medication beliefs (Selinger et al., 2013). Thus, theoretical models such as the NCF (Horne & Weinman, 2002) can be used to specify where to target intervention strategies within adherence interventions. To develop effective interventions, it is essential to understand adolescent’s adherence not only in terms of medication taking, but also in terms of broader behaviours such as attending medical appointments and making lifestyle behaviour changes. The aim of this research was to explore the experience and support needs of adolescents with IBD to facilitate optimum treatment adherence.

## Method

### Design

The study employed a qualitative approach, using semi-structured interviews and arts-based methods. This qualitative design facilitated in-depth investigation of participants’ lived experience of adhering to their IBD treatment and their views on adherence support needs (Ormston et al., 2014).

### Sample and recruitment

Purposive and opportunistic sampling was used to identify adolescents (aged 13–18) diagnosed with IBD, parents/carers of adolescents (aged 13–18) with IBD and healthcare professionals who work with adolescents with IBD. Healthcare professionals were classified either as medical professionals (e.g. doctors, nurses) or allied health professionals (e.g. psychologists, dieticians). Participants were recruited via a large UK-based Children’s Hospital and social media pages of relevant charities (Crohn's and Colitis UK), using study advertisements and in-clinic invitations. Those interested in the research were provided with a digital study information pack including information sheet, consent form and demographic sheet. A favourable ethical opinion was obtained from an NHS Research Ethics Committee (IRAS #268658) and University Research Ethics Committee (ref: #1648).

### Data collection

Qualitative data were collected by CS between 2020 and 2021. Interviews were conducted via telephone or videocall (Zoom), were audio-recorded and lasted approximately 60 min. Questions explored participants’ views and experiences of the barriers and facilitators adolescents faced in managing their IBD and adhering to their treatment plan, as well as how an intervention could improve adolescents’ adherence. Topics included family’s medication and illness beliefs as well as practical, social, and emotional challenges of adherence. To facilitate data collection, adolescents and parents were invited to complete a creative mapping task. This involved participants identifying their most significant adherence barrier and then arranging possible intervention strategies for overcoming this barrier on a template ‘map’ categorising support options as ‘very helpful’, ‘slightly helpful’, or ‘unhelpful’(see supplementary file 1).

As a token of thanks, parents and adolescents were compensated for their participation with a £15 online shopping voucher.

### Data analysis

Interviews were recorded, transcribed and anonymised. Transcripts were analysed using thematic framework analysis (Ritchie & Spencer, 1994), an approach which supports identification, analysis and reporting of patterns within the data. Framework analysis was well suited to the project owing to its methodological flexibility in comparing experiences both within, as well as across cases (Gale et al., 2013). Data analysis was carried out in accordance with the five stages of the framework method (Ritchie & Spencer, 1994). First, researchers became familiar with the data through repeated reading. Transcripts were then coded by assigning labels to salient pieces of data, leading to the creation of a thematic coding framework. The coding framework was applied to the whole data set before comparisons were made between participant groups. Finally, interpretative analyses were undertaken, moving beyond descriptive themes. A subset of transcripts was independently coded by two members of the research team (CS and GH/LA/RS). Qualitative analysis of the creative task outputs resulted in a list of intervention priorities and strategies.

## Findings

### Participants

Thirty-three interviews were conducted, with adolescents with IBD (*n* = 12), parents of adolescents with IBD (*n* = 13) and healthcare professionals (*n* = 8) who work with adolescents with IBD (Tables 1 and 2). The sample included nine parents whose children had also partaken in the research; however, all parents and adolescents were interviewed separately.

**Table 1.** Participant characteristics.

	Adolescents (*N* = 12)	Parents (*N* = 13)
Age (mean, SD)	14.75 (1.42)	49.08 (5.6)
Gender		
Male	7	2
Female	5	11
Ethnicity		
White British	9	11
White Irish	1	
Indian	1	1
Pakistani	1	1
Adolescent age at diagnosis (mean, SD)	12.08 (2.66)	10.77 (3.26)
Adolescent diagnosis		
Crohn’s disease	11	9
Ulcerative colitis	1	3
IBD unclassified		1
Adolescent medication		
Azathioprine	5	5
Humira	5	6
Infliximab	2	1
Methotrexate	2	
Ustekinumab	1

**Table 2.** Healthcare professional’s job roles.

Pseudonym	Job role
Healthcare professional 1	Medical professional
Healthcare professional 2	Medical professional
Healthcare professional 3	Medical professional
Healthcare professional 4	Medical professional
Healthcare professional 5	Allied healthcare professional
Healthcare professional 6	Allied healthcare professional
Healthcare professional 7	Allied healthcare professional
Healthcare professional 8	Medical professional

### Thematic findings

Analysis generated three themes exploring the experience and support needs of adolescents with IBD to facilitate optimum treatment adherence: (1) Striving for normality; (2) taking responsibility for IBD management; (3) seeking supportive environments.

#### Striving for normality

Adolescents wanted to live a ‘normal’ life, free from the restrictions of living with IBD. Often this ‘striving for normality’ provided motivation for adolescents to follow their treatment plan; doing so was perceived to encourage wellness and remission. Remission was constructed as the goal for all families, referred to as a ‘golden ticket’ (Maxine, parent). Families discussed remission as if it were a cure for IBD, rather than a temporary state of symptom reduction. Despite still experiencing some IBD symptoms, adolescents experiencing remission suggested they were able to ‘look past my medical limitations and do what I wanna do’ (Damien, young person, aged 16, Crohn’s Disease).

IBD medications were therefore perceived as the gateway to remission, motivating adolescents to maintain adherence both before and during a period of remission to ‘take care of themselves so they can get better in the future’ (Maria, young person, aged 14, Ulcerative Colitis); such motivation was maintained even when the medications prescribed were perceived ineffective or unpleasant.

The idea of going into remission and being able to lead a normal life and all I have to do is take a medication that may be a bit inconvenient and may not taste nice … and you can finally have a healthy body (Damien, young person, aged 16, Crohn’s Disease)

Parents were concerned that adolescents did not understand the importance of continued adherence during periods of wellness, however, and often took it upon themselves to provide frequent reminders. For adolescents, their parents’ persistent hypervigilance and medication prompts were a constant reminder of the chronicity of IBD, which contrasted with the normality they felt or sought. These conflicting views often promoted tension in the home, which in turn encouraged adolescents to display volitional non-adherence.

My parents were like every day, have you taken it, have you taken it? … it did start to annoy me and I thought you know what, I might as well not take it and umm see what happens (Erika, young person, aged 15, Crohn’s Disease)

Parents further displayed hypervigilance by checking for indications of an IBD flare, seeking to contain their own worry about future adverse events: ‘I am just constantly checking that [they’re] OK daily … check [their] poo when [they] go to the toilet’ (Phillipa, parent). Adolescents perceived this to be intrusive and adversely impacted their desire to be ‘normal’.

For adolescents, hospital appointments were another unwanted reminder that they lived with a chronic condition. Overall, interactions within hospital settings such as undergoing medical procedures and attending pre-appointment blood tests were experienced as disruptive for adolescents. However, some adolescents were able to make appointments with nurses who visited the family’s home which was well received.

I think for adolescents [hospitals] are quite frightening … there was a lot of people with tubes, NG [Naso-gastric] tubes and things like that … , when [my child] went in for his colonoscopy there was lots of very sick children, and I guess he was sort of thinking, crikey, you know, I could be like that (Phillipa, parent)

Physical and cognitive fatigue as well as persistent pain and lack of control over their bowels featured heavily in adolescents’ experiences. As such, their accounts were saturated with examples of feeling unable to participate in ‘normal’ social activities. Adherence then was constructed as having to engage with behaviours that made adolescents stand out from their peers, which, in turn, then hindered adherence. Lifestyle factors (diet, exercise, sleep) were most vulnerable to non-adherence during social situations, for example, when spending time with friends or whilst on holiday. Prolonged adherence to medications with severe side effects was challenging for adolescents, as they perceived their treatment to shrink their world rather than open it up. The persistent need to cancel social events was difficult for friendship groups to understand, leaving adolescents feeling socially isolated and questioning the benefits of adhering to their treatment.

I feel like [my medication] really limits me on what I can do in terms of going out and hanging with my friends (Leila, young person, aged 17, Crohn’s Disease)

To minimise feelings of difference, adolescents avoid disclosing IBD treatment routines with their friends. Therefore, treatments that were perceived to be easy and discrete to administer, were preferred due to their reduced social impact;

The medication I have … you could just cut out a couple and just put them in your pocket, when you’re going out (Felix, young person, aged 13, Crohn’s Disease).

#### Taking responsibility for IBD management

To manage their IBD, families and healthcare professionals described a need for adolescents to take responsibility for their condition by establishing and following daily treatment routines. However, parents aimed to relieve this burden by retaining control.

### Establishing and following treatment routines

While adolescents valued being included in the construction of their treatment plans, responsibility for making key decisions was experienced as overwhelming. Subsequently, adolescents and their caregivers, relied on healthcare professionals to make such decisions, which could have clinical consequences.

You discuss [their treatment] with them and you give them the choice … in my experience most of the time families get confused, they say doctor you decide (Healthcare professional 3, Medical Professional)

In addition, training facilitated by IBD nurses, which allowed adolescents to learn how to administer medications safely at home, was well received by adolescents as injectable medications were ‘quite easy to mess up’ (Vihaan, young person, aged 14, Crohn’s Disease). Learning the process of injecting medications was emotionally challenging for some adolescents as they feared the process going wrong. Parents also expressed concerns over causing harm to their child and when parents were anxious about medication, such emotions were transferred to the young person.

[parents] feeling nervous and like kind of saying ooo I don’t want you to do this because it’s going to hurt you or something like that maybe might make you back off and make you think it’s going to be bad for you (Amy, young person, aged 14, Crohn’s Disease)

Implementing treatment routines initially involved adolescents understanding the process of how to follow IBD treatment plans; this was often difficult when adolescents were prescribed multiple medications. Most adolescents aimed to identify an appropriate time to take their medication within their normal routine. The use of self-implemented treat-based rewards facilitated treatment adherence as did improvements in clinical results. However, adolescents’ ability to remember their treatment plan was often threatened during times when family routines were disrupted (e.g. school holidays or weekends). Equally, medications were often forgotten due to being busy with educational or social activities; ‘sometimes like you're busy at night and you just forget, and then you’re just tired and fall asleep’ (Jordan, young person, aged 17, Crohn’s Disease). To prompt adherence, some adolescents placed their medications in a clearly visible location. Whilst most visual prompts were implemented by parents, adolescents preferred to have some autonomy. To overcome medication forgetfulness, digital reminders were often used.

I’ve got my phone that tells me, I’ve got my mom which is most and then I’ve got my smart speaker. And without all those I probably wouldn’t know what medication is (Felix, young person, aged 13, Crohn’s Disease)

As their condition improved, most adolescents received advice from healthcare professionals on lifestyle behaviours (diet, exercise, sleep). When adopted into their treatment routine, adolescents noticed further improvement to their health; ‘since I have been like doing more exercise, I feel like [my IBD] is more controlled’ (Maria, young person, aged 14, Ulcerative Colitis). Families and healthcare professionals discussed the importance of including individualised dietary advice in adolescents’ IBD treatment plan. However, some parents strongly refuted the role of diet in the management of their child’s IBD, due to misinformation claiming diet can both cause and cure IBD. To avoid feelings of blame, some parents refuted any associations with diet in their child’s treatment plan; ‘then you live with that guilt that you’ve done something or given something to your child that has caused this disease.’ (Maxine, parent).

### Absorbing burden

Parents felt responsible for shielding their child from the burden of managing their IBD. For some parents, this involved taking over full management of their child’s condition, including arranging medical appointments and ordering medical supplies. Whilst ‘it was a strain, having to do that regularly’ (Julie, parent), this was perceived as part of the normal care parents should provide for their child.

He has blood tests every three months, so I book all of that. He wouldn't have a clue when he's blood tests are due (Philippa, parent)

Philippa went on to elaborate how their child was unable to manage their condition without parental support, ‘I don't feel he's ready to look after his own IBD at the moment, even though he's [in his late teens] he probably thinks he can, but I don't think he can’. This was commonly described by parents who felt their child was too young to look after their health independently, despite adolescents requesting more self-management responsibilities. However, removing condition management behaviours from adolescents’ lives prevented the development of essential self-management behaviours.

If my mum said to me Aiden, I want you umm, I’m not going to put [your medication] out for you anymore … I feel like I could (Aiden, young person, aged 15, Crohn’s Disease).

#### Seeking supportive environments

IBD was understood to be an uncomfortable and stigmatised condition, which frequently hindered adolescents’ adherence. Adolescents therefore sought supportive environments free of stigmatisation.

Parents discussed the taboo of discussing bowel-related symptoms and the impact this had on adolescents’ emotional wellbeing:

I wish there was the confidence to, you kinda need to make shit sexy, literally … People don’t like talking about bowel movements and farts (Sadie, parent).

To avoid stigmatisation and cruel comments, adolescents often concealed their IBD from others; ‘it’s not something I want the whole world to know’ (Vihann, young person, aged 14 Crohn’s Disease). However, when their IBD could not be hidden, adolescents experienced hurtful comments from others; often targeted at IBD treatment routines, which encouraged non-adherent behaviours.

I remember taking the liquid diet … someone threw some chicken at me and they were going urgh what are you eating an ice lolly for you weirdo … it made me feel really bad like eating an ice-cream at school for a few weeks … I was worried what people would think … I used to be really hungry, but I didn’t want to [eat] just in case people thought I was weird (Erika, young person, aged 15, Crohn’s Disease)

Encouraging adolescents to view their condition and treatment routine as a welcomed part of normal life was perceived to be beneficial for their emotional wellbeing, as parents believed this would support their child to develop a healthy body image and develop the confidence to discuss their IBD with others. Within the home, parents aimed to include the child’s IBD in conversations to reduce feelings of embarrassment and maintain their treatment adherence. Adolescents were grateful for their supportive home environments. For some parents, the provision of persistent emotional and practical support was viewed as part of parents’ identity.

My role as a dad … as a father I am there, like you know she can tell me anything she wants. I tell her not to keep it bottled up and if she needs you know someone to talk to even as a friend, so I tell her don’t see me as a dad, you can see me as a friend as well (Hasim, parent)

Joining IBD social media groups allowed adolescents to build a network of support inclusive of those who were perceived to ‘know exactly what you are going through’ (Laura, young person, aged 16, Crohn’s Disease). Group members could also provide a sense of acceptance over living with IBD. The nature of online communication further encouraged adolescents to reach out for support when they needed it. Adolescents often reviewed their condition knowledge harshly and felt that they were ill informed in comparison to other adolescents with IBD. This was sometimes the result of parents withholding potentially frightening health information from their child. Online support networks were perceived as a reliable trustworthy resource to further adolescents’ knowledge and understanding of their treatment adherence behaviours. Online support groups encouraged a selection of adolescents to feel able to share their diagnosis with friendship groups and in doing so, received practical and emotional support from their friends, who were willing to adapt their normal activities to be inclusive of the young person’s health needs.

They used to always come to the toilet with me and be like oh no it’s ok I’ll wait for you (Erika, young person, aged 15, Crohn’s Disease)

Forming an honest relationship between adolescents and healthcare professionals promoted treatment adherence behaviours. Gastroenterology healthcare professionals demonstrated understanding and empathy over adolescents’ experiences and were perceived to be a credible source of information due to their IBD expertise; ‘They are professionals they know what they're doing’ (Jordan, young person, aged 17, Crohn’s Disease). Healthcare professionals further extended the trusting relationship by encouraging honesty from adolescents about their adherence behaviours. The relaxed tonality within these conversations allowed adolescents to discuss changes to their health and share potentially uncomfortable omissions of non-adherent behaviours. By doing so, adolescents were developing essential self-management behaviours as well as providing healthcare professionals with valuable information on how to amend treatment plans to meet adolescents’ needs. Healthcare professionals’ inclusive approach further empowered adolescents to feel able to discuss their IBD.

I’m not just a kid anymore like I know I am more independent about it and I can like figure it out more myself like if [the consultant] has a question, he will always ask me … I think that’s quite good (Amy, young person, aged 14, Crohn’s Disease)

## Priorities for supporting treatment adherence

**Figure 1.**
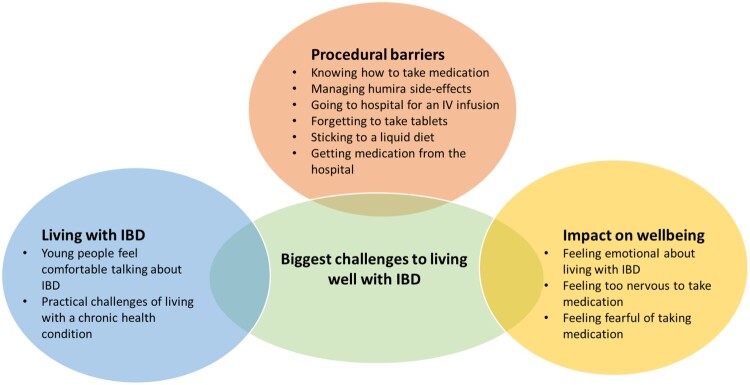
Barriers identified within the creative task.

Adherence barriers identified through the creative mapping task included: challenges of living with IBD, how and when to take specific medications and impact of performing self-management behaviours on adolescents’ emotional wellbeing (Figure 1). Participants’ self-generated ‘helpful’ or ‘very helpful’ intervention strategies to overcome these barriers indicated a need to facilitate young person autonomy alongside practical and emotional support from parents/guardians. Misinformation and parental ‘nagging’ were considered ‘unhelpful’ for overcoming adherence barriers. Synthesising findings from the creative mapping task with themes from interviews led generated seven priorities for supporting adolescents’ IBD treatment adherence (Table 3).

**Table 3.** Priorities for supporting adolescents’ IBD treatment adherence.

Priority	Adherence support need	Intervention strategy
1	Adolescents need support from understanding others, who can provide helpful adherence advice.	Facilitating social support opportunities, (either face-to-face or through videos/podcasts) where adolescents can receive support from other adolescents living with IBD.
2	Adolescents perceived parental support could sufficiently overcome their adherence barriers, without the need to perform autonomous self-management behaviours.	Future interventions should be inclusive of parental support. Furthermore, interventions should look to encourage the transferral of self-management skills from parents to adolescents, to support the development of autonomous self-management behaviours.
3	Adolescents choose not to adhere to their treatment plan due to parental behaviours such as ‘nagging’, a lack of empathy, or parental anxiety.	Interventions targeting parental behaviour and wellbeing could be highly beneficial for adolescents’ treatment adherence behaviours.
4	Adolescents need further IBD education from credible sources, in the forms of leaflets, videos, and websites.	Creation of multi-media educational resources on a range of relevant IBD topics. Such resources should be co-designed with adolescents to ensure they are appropriate for the target population. Co-writing the information with healthcare professionals will further ensure credibility of the produced educational resources.
5	Disclosure preferences determine the extent to which peer support is beneficial for adolescents.	Tailored guidance and training on how to discuss IBD and health needs with others can be provided either face-to-face or remotely.
6	Adolescents want healthcare professionals to provide emotional and practical support to overcome adherence barriers within consultations.	Additional training for healthcare professional, addressing adolescents’ additional adherence support needs, can be delivered face-to-face or remotely (e.g. webinars).
7	Holding an optimistic view of the future can encourage adolescents’ adherence.	Social support strategies (either face-to-face or videos/podcasts) can be utilised to encourage adolescents to view treatment adherence as a gateway to remission and/or other positive future experiences.

## Discussion

Three key themes described adolescents’ IBD treatment adherence experiences and support needs: (1) striving for normality, (2) taking responsibility for IBD management and (3) seeking supportive environments. Findings suggested adherence behaviours for adolescents varied in response to complex situational factors, including where the behaviour needed to be performed and the presence of others while performing the behaviour.

Families discussed a cyclical experience of striving for normality while adapting to the abnormality of IBD. Adhering to treatment plans in social situations was often disliked by adolescents, who wanted to preserve privacy over their condition. Existing research shows that while adolescents may fear disclosing IBD to friends, they often encounter positive interactions when this information is shared (Carter et al., 2020). Providing guidance on how adolescents can successfully facilitate conversations about their IBD with peers is therefore warranted.

Experiencing cruel or misinformed comments due to the stigmatisation of IBD, also impacted adolescents’ confidence and wellbeing, often promoting non-adherence. Experiencing IBD stigma has previously been shown in adolescents and adults to lead to social isolation, increased depression and anxiety, poorer self-esteem, lower health-related QoL (HrQoL) and reduced treatment adherence (Gamwell et al., 2018; Taft et al., 2009). The experiences discussed by adolescents in this study highlight a need for intervention strategies that specifically assist young people in coping with IBD stigma.

Adolescents held remission in high regard and viewed this as the gateway to living a ‘normal’ life. However, like most people with IBD (including adults), many adolescents continued to experience some IBD symptoms during periods of remission, all be it at a lesser severity (Bielefeldt et al., 2009; Gong et al., 2021). Kitchen et al. (2020) identified variance in young people’s understanding and definition of IBD remission. The current study’s findings support this, emphasising a need to ensure adolescents’ and families’ remission expectations are realistic, to prevent feelings of disappointment and volitional non-adherence during periods of ‘wellness’.

In our research, parents constructed themselves as responsible for the management of their child’s condition. However, ‘over-protective’ parenting behaviours were shown here (and by others) to negatively impact adolescents’ acquisition of essential self-management skills. Adolescents wanted to gain more autonomy and feeling hindered to do this resulted in volitional non-adherence and tension within the home. Greater family conflict is associated with poor adherence behaviours for a range of chronic health conditions (Psihogios et al., 2019). By providing parental support to reduce hypervigilance it may be possible to facilitate transfer of self-management responsibilities to adolescents, supporting them to manage their condition independently. Trusted relationships with healthcare professionals may be important here by providing a space for adolescents to discuss their condition and treatment adherence openly and confidently.

Implementing treatment routines within the home was sometimes challenging; most adolescents discussed forgetting their medication, often due to changing routines or other distractions. This was unsurprising as forgetfulness has previously been identified as a common treatment adherence barrier for this population (Gray et al., 2012; Schurman et al., 2011). Families often implemented strategies to prompt adherence such as digital reminders and visual or verbal prompts. Strategies that promoted a sense of normality and autonomy were often preferred by adolescents. Therefore, a tailored approach may be beneficial when providing adolescents with adherence behaviour support, to identify strategies and techniques that meet an individual’s preferences and needs.

Adolescents discussed wanting to know more about IBD and the medications they were taking; however, the articulate way adolescents discussed their health is suggestive of a perceived rather than actual deficit in knowledge. Fishman et al. (2011) also identified high levels of IBD general knowledge in adolescents. The current research acknowledged a conscious effort to shield adolescents from unpleasant information; whilst potentially frightening, an awareness of such possibilities is important for the self-management of IBD in adulthood. To improve treatment adherence behaviours, adolescents may benefit from materials which can confirm and/or enhance their IBD knowledge and subsequently increase their self-management self-efficacy. Following Social Cognitive Theory (Bandura, 1977), self-efficacy has previously been shown to predict medication adherence in a wide range of chronic health conditions (Holmes et al., 2014), including IBD (Cook et al., 2010).

Consistent with the Necessity and Concerns Framework (Horne & Weinman, 2002), when adolescents perceived adherence as necessary (e.g. for achieving remission), and when they had few concerns about doing so, adherence increased. However, when adolescents felt anxious about following their treatment plan, most commonly in social situations, these concerns led to non-adherence. Volitional non-adherence was also reported when adolescents felt there was little need to follow either part or the whole of their treatment plan and felt unconcerned about non-adherence. Medication beliefs have previously been identified as a significant cause of non-adherence in adults with IBD (Jackson et al., 2010). The perceived necessity to take IBD medication may change during an IBD flare, as experiencing symptom relief outweighs an individuals’ medication concerns (Hall et al., 2007; Moshkovska et al., 2009).

## Implications for practice

The research highlights several implications for practice, including the need for future interventions to: (1) target adolescents’ confidence to follow the entirety of their treatment plans, (2) deliver guidance and support on how to perform autonomous IBD self-management behaviours, (3) offer social support to promote a sense of normality in living with IBD and (4) provide trustworthy information to enhance adolescents’ understanding of their IBD**.**

## Strengths and limitations

To our knowledge, this is the first study to explore the experience and support needs of adolescents in adhering to their whole IBD treatment plan (i.e. including medication as well as lifestyle behaviour modifications, appointment attendance). Findings are instrumental for the development of interventions for improving adherence in this group. The study had a varied sample, including those living with Crohn’s Disease, Ulcerative Colitis and IBD Unclassified. The inclusion of nine families allowed for comparisons to be directly made between treatment adherence barriers and facilitators within the same household. However, the sample was limited in terms of ethnic diversity. Previous research has identified individuals of Indian ethnicity are more likely to experience greater IBD disease severity (Misra et al., 2019). Future research may look to utilise stratified sampling methods to ensure a wider range of ethnicities are included. While families were predominantly sampled from those with adolescents whose IBD was well controlled, healthcare professionals were able to provide insights on adolescents who presented in the clinic with persistent poor management. Most interviews with parents and adolescents were conducted during the initial months of the Covid-19 pandemic, therefore the immediate and long-term disruptions caused by Covid-19 are may have impacted the data.

## Conclusion

There is no single behaviour or set of behaviours that facilitate optimum treatment adherence. Rather, adolescents shared a range of complex and situational factors driving their adherence behaviours. The adherence barriers and facilitators identified within this research can be utilised to develop an evidence-based theory-driven treatment adherence intervention that is effective for adolescents with IBD.

## Author’s contribution

Dr Cassandra Screti: Investigation, Formal Analysis, Writing – Original Draft, Writing – Review and Editing. Dr Lou Atkinson: Formal Analysis, Writing – Review and Editing, Supervision. Prof Rachel Shaw: Formal Analysis, Writing – Review and Editing, Supervision. Dr Rafeeq Muhammed: Writing – Review and Editing. Dr Gemma Heath: Conceptualisation, Formal Analysis, Writing – Original Draft, Writing – Review and Editing, Funding acquisition, Supervision.

## Supplementary Material

Supplementary File 1.docx

## Data Availability

Anonymised data that support the findings of this study are available on request from the corresponding author 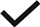 (a) Institutional Review Board Statement: The study was conducted in accordance with the Declaration of Helsinki and was approved by an Institutional Review Board/Ethics committee. See details under Methods. (b) The study received an exemption from an Institutional Review Board/Ethics committee; See details under Methods.
