# Supplementary material for: ‘We need to make “shit” sexy’ a qualitative study exploring treatment adherence in adolescents with inflammatory bowel disease
Source: Health Psychol Behav Med. 2025 May 5;13(1):2500323. doi: 10.1080/21642850.2025.2500323 (PMC12057774; doi:10.1080/21642850.2025.2500323)
Supplement: Supplementary File 1.docx [file RHPB_A_2500323_SM4981.docx]

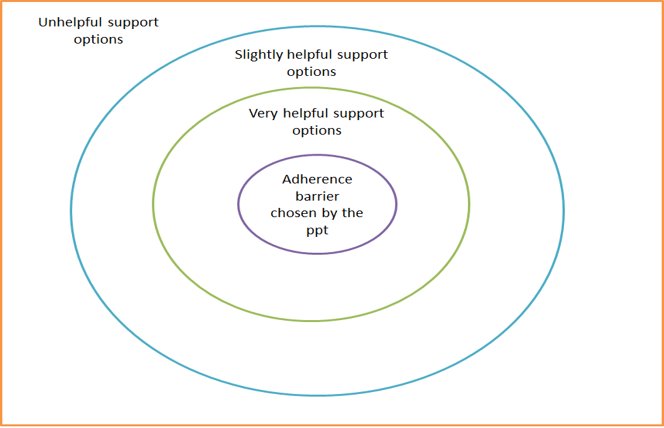
Supplementary file 1: Example of the creative task


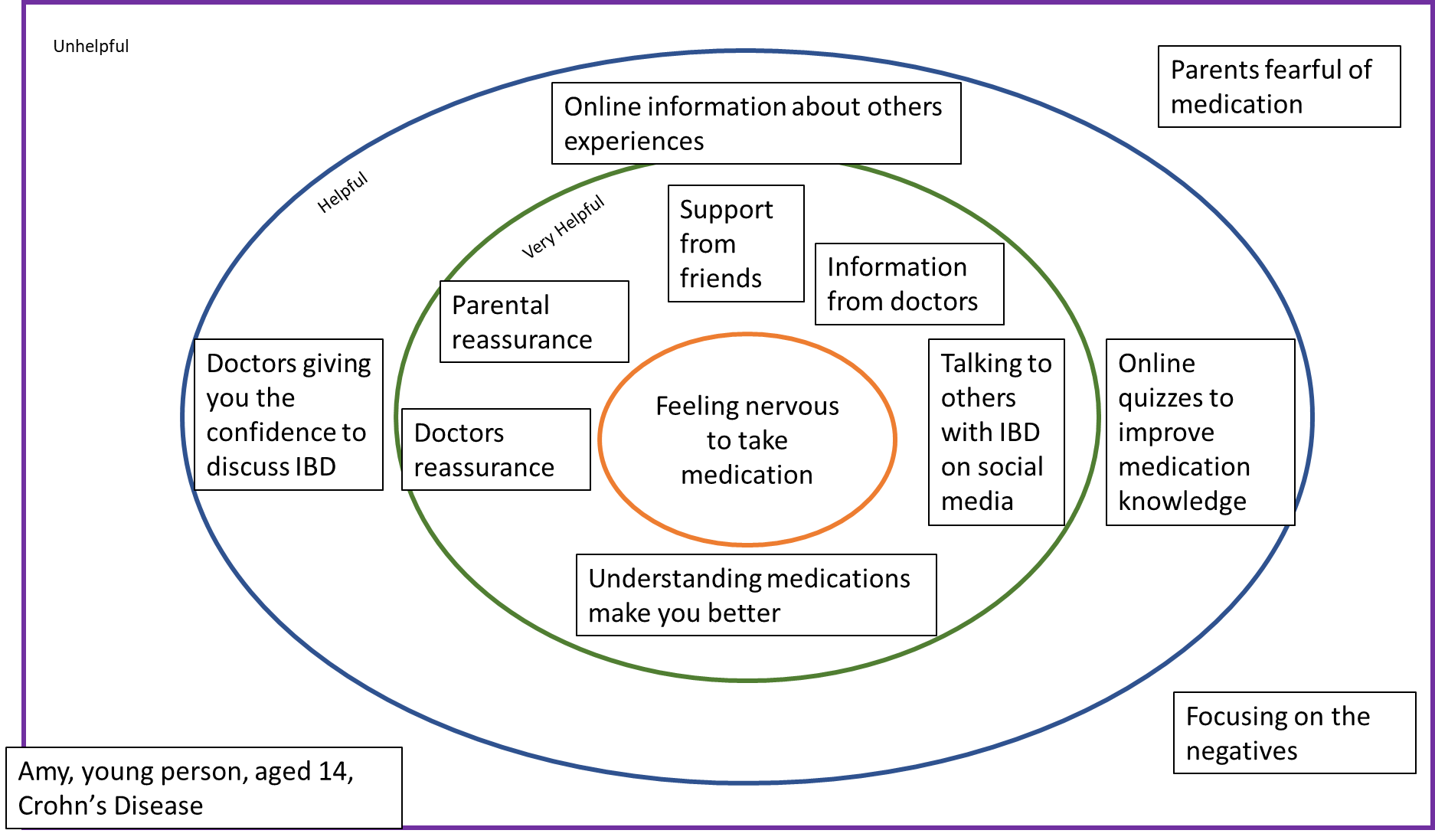


Supplementary file 1 Long Description: The creative task template (top) and an example as completed by Amy; a young person aged 14 with Crohn’s Disease. A large rectangle fills the page and includes Amy’s suggestions for unhelpful options to overcome the barrier of feeling nervous to take medication. These unhelpful solutions include focusing on the negatives and parents being fearful of medications. Within the rectangle is a large oval, containing solutions viewed as helpful including: online information about other’s experiences, doctors giving you the confidence to discuss IBD, and online quizzes to improve medication knowledge. Within this oval is a smaller circle containing the participant’s self-generated very helpful solutions including support from friends, parental reassurance, information from doctors, talking to others with IBD on social media, understanding medications make you better and doctors’ reassurance. In the centre of the smaller circle is an oval containing the participant’s chosen barrier of feeling nervous to take medication.
